# CyclosporinA Derivative as Therapeutic Candidate for Alport Syndrome by Inducing Mutant Type IV Collagen Secretion

**DOI:** 10.34067/KID.0000000000000134

**Published:** 2023-05-05

**Authors:** Jun Kuwazuru, Mary Ann Suico, Kohei Omachi, Haruka Kojima, Misato Kamura, Shota Kaseda, Teppei Kawahara, Yuki Hitora, Hikaru Kato, Sachiko Tsukamoto, Mikiyo Wada, Toshifumi Asano, Shunsuke Kotani, Makoto Nakajima, Shogo Misumi, Yuya Sannomiya, Jun Horizono, Yuimi Koyama, Aimi Owaki, Tsuyoshi Shuto, Hirofumi Kai

**Affiliations:** 1 Department of Molecular Medicine, Graduate School of Pharmaceutical Sciences, Kumamoto University, Kumamoto, Japan; 2 Global Center for Natural Resources Sciences, Faculty of Life Sciences, Kumamoto University, Kumamoto, Japan; 3 Department of Instrumental Analysis, Graduate School of Pharmaceutical Sciences, Kumamoto University, Kumamoto, Japan; 4 Useful and Unique Natural Products for Drug Discovery and Development (UpRod), Program for Building Regional Innovation Ecosystems, Kumamoto University, Kumamoto, Japan; 5 Department of Natural Medicines, Graduate School of Pharmaceutical Sciences, Kumamoto University, Kumamoto, Japan; 6 Department of Organic Chemistry, Graduate School of Pharmaceutical Sciences, Kumamoto University, Kumamoto, Japan; 7 Department of Environmental and Molecular Health Sciences, Graduate School of Pharmaceutical Sciences, Kumamoto University, Kumamoto, Japan

**Keywords:** CKD, alisporivir, Alport syndrome, basic science, collagen trimer, cyclophilin, cyclosporin A, type IV collagen

## Abstract

**Key Points:**

- Screening of natural product extracts to find candidate compounds that increase mutant type IV collagen *α*3,4,5 (*α*345(IV)) trimer secretion in Alport syndrome (AS).
- Cyclosporin A (CsA) and alisporivir (ALV) increase mutant *α*345(IV) trimer secretion in AS.
- PPIF/cyclophilin D mediates the effect of CsA and ALV on mutant trimer secretion.

**Background:**

Type IV collagen *α*3,4,5 (*α*345(IV)) is an obligate trimer that is secreted to form a collagen network, which is the structural foundation of basement membrane. Mutation in one of the genes (*COL4A3*, *A4*, *A5*) encoding these proteins underlies the progressive genetic nephropathy Alport syndrome (AS) due to deficiency in trimerization and/or secretion of the *α*345(IV) trimer. Thus, improving mutant *α*345(IV) trimerization and secretion could be a good therapeutic approach for AS.

**Methods:**

Using the nanoluciferase-based platform that we previously developed to detect *α*345(IV) formation and secretion in HEK293T cells, we screened libraries of natural product extracts and compounds to find a candidate compound capable of increasing mutant *α*345(IV) secretion.

**Results:**

The screening of >13,000 extracts and >600 compounds revealed that cyclosporin A (CsA) increased the secretion of mutant *α*345(IV)-G1244D. To elucidate the mechanism of the effect of CsA, we evaluated CsA derivatives with different ability to bind to calcineurin (Cn) and cyclophilin (Cyp). Alisporivir (ALV), which binds to Cyp but not to Cn, increased the trimer secretion of mutant *α*345(IV). Knockdown studies on Cyps showed that PPIF/cyclophilin D was involved in the trimer secretion-enhancing activity of CsA and ALV. We confirmed that other *α*345(IV) mutants are also responsive to CsA and ALV.

**Conclusions:**

CsA was previously reported to improve proteinuria in patients with AS, but owing to its nephrotoxic effect, CsA is not recommended for treatment in patients with AS. Our data raise the possibility that ALV could be a safer option than CsA. This study provides a novel therapeutic candidate for AS with an innovative mechanism of action and reveals an aspect of the intracellular regulatory mechanism of *α*345(IV) that was previously unexplored.

## Graphical abstract

**Figure:**
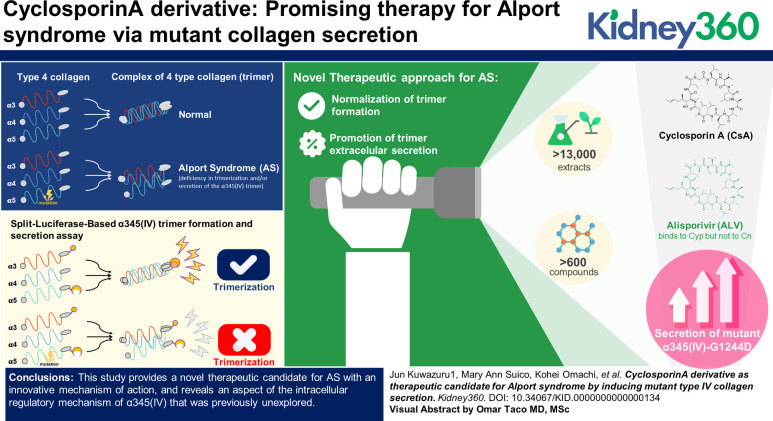

## Introduction

Alport syndrome (AS) is a genetic disease of progressive glomerulonephritis caused by mutation in one of the type IV collagen genes (*COL4A3*, *A4*, or *A5*) that compose the glomerular basement membrane (GBM).^1,2^ Type IV collagen forms intracellular *α*3/*α*4/*α*5(IV) subunits to produce type IV collagen *α*3,4,5 (*α*345(IV)) trimers, which are secreted from the cells to polymerize and become part of the GBM. Genetic mutation in any of the *COL4A3/4/5* genes leads to failure in trimer formation, thinning of the GBM, disruption of the podocyte scaffold structure, and subsequent proteinuria.^3^ Comprehensive studies on AS had yielded much better understanding of this disease and more knowledge on the correlation between genotype and clinical phenotypes.^4–6^ AS has a wide variety of mutations, of which the missense mutation is most frequently reported.^7–9^ Among missense mutations, the severity of the disease varies depending on the mutation. It has been suggested that the phenotype is milder if the extracellular secretion of *α*345(IV) trimer is higher.^10^ Clinical reports showed that patients with even a small level of *α*5(IV) staining in the GBM have better prognosis than those with no staining at all.^11,12^ On the basis of these studies, normalization of trimer formation and promotion of trimer extracellular secretion could be a promising therapeutic approach for AS. To date, there is a lack of therapeutic drugs targeting the causative protein, that is, mutant type IV collagen.

We previously constructed an evaluation system for *α*345(IV) trimer formation and secretion.^13^ This system is based on the fusion of split nanoluciferase fragments, such as small binary technology (SmBiT) and large BiT (LgBiT), which were tagged at C-terminal of *α*3(IV) and *α*5(IV) chains, respectively. Trimerization in the presence of *α*4(IV) fuses these fragments to produce quantifiable luminescence.^13^ Here, we used this system to screen original natural product extract libraries generated in Kumamoto University with the aim of finding therapeutic agents targeting the *α*345(IV) trimer. The screening of >13,000 extracts and >600 compounds yielded cyclosporin A (CsA) as the only compound that increased the secretion of mutant trimer. Importantly, analysis of structure–activity relationship revealed that alisporivir (ALV), a CsA derivative, worked similarly as CsA. Mechanistically, we identified the involvement of cyclophilin D (CypD), encoded by *PPIF* gene,^14^ in the secretion-enhancing effect of CsA and ALV. CsA was reported to improve proteinuria and delay the progression of AS in clinical studies but concerns about its nephrotoxicity still preclude its wide use in the clinic for patients with AS patients.^15,16^ On the other hand, ALV has a better safety profile than CsA.^17^ Our study suggests that ALV could be a better candidate for clinical use than CsA and reveals new mechanistic insights in the modulation of mutant trimer secretion.

## Materials and Methods

### Plasmids and Reagents

The preparation of the plasmids for human *α*3(IV), *α*4(IV), and *α*5(IV) for nanoluciferase complementation system was previously described.^13^ In brief, the SmBiT and LgBiT fragments were fused to the C-terminal of *α*3(IV) and *α*5(IV) in the pFC36K SmBiT TK-neo Flexi and pFC34K LgBiT TK-Neo Flexi vectors (Promega), respectively. *α*4(IV) was subcloned into pEB multi-Hygromycin vector (Wako) and fused with 3×FLAG tag. Mutants of *α*3(IV) and *α*5(IV) were generated by site-directed mutagenesis as previously described.^10,13^ The plasmid for PPIF/CypD/CypF (HG14689-CM) was from Sino Biological. For stable cell lines, *α*3(IV)-SmBiT, *α*4(IV)-3×FLAG, and *α*5(IV)-LgBiT wild type or G1244D were subcloned into pLVSIN vector (Takara), respectively, containing hygromycin, puromycin, or blasticidin resistance genes. The reagents CsA, PSC-833, and trimethyl amine *N*-oxide (TMAO) were from Sigma-Aldrich. Dimethyl sulfoxide, glucose, mannitol, sorbitol, sucrose, and taurine were from Nacalai Tesque. Trehalose was from Tokyo Chemical Industry. Brefeldin A (BFA) was from calbiochem. ALV (HY-12559) was obtained from Medchem Express. FK506/Tacrolimus was from Abcam.

### Cell Culture and Establishment of Stable Cell Line

HEK293T cells (ATCC, VA) were maintained in Dulbecco Modified Eagle Medium (DMEM) supplemented with 10% FBS and 100-U penicillin and streptomycin. We used lentiviral system to create stable cell lines, cotransfecting pCMV-R8.74 (Addgene 22036), pMD2.G (Addgene 12259), and each tagged *α*3(IV), *α*4(IV), and *α*5(IV) pLVSIN plasmids in HEK293T cells using TransIT-LT1 (Mirus). Twenty-four hours post-transfection, the medium was replaced with virus collection medium and incubated for 24 hours. The pooled medium from two rounds of virus collection was passed through 45-*μ*m filter. Viral particles were concentrated using Lenti-X Concentrator, centrifuged, and resuspended in phosphate buffered saline. Viral particles of *α*4(IV)-FLAG, *α*3(IV)-SmBiT, and *α*5(IV)-LgBiT were sequentially transduced in HEK293T cells. The cell lines stably expressing *α*345(IV) trimer were generated by antibiotic selection.

### Screening

The *α*345(IV)-G1244D stable cell line was used for screening the Prestwick phytochemical library (GreenPharma), and Kumamoto University's collection of natural product libraries named Useful and Unique Natural Products for Drug Discovery (UpRod), which is a proprietary material generously provided by Kumamoto University UpRod Program. The libraries consist of extracts from marine sponges, fungi, bacteria, and plants. For screening, cells were plated on coated 96-well white/clear bottom plate (corning) in phenol red-free DMEM.^13^ Subconfluent cells were treated with 10-*μ*M compound or 10 *μ*g/ml extract in medium containing 200 *μ*M L-ascorbic acid for 24 hours. Culture media were collected and assayed for nanoluciferase activity using Nano-Glo Live Cell Assay reagent and GloMax Navigator system (Promega).

### Transfection, Treatment, and Luciferase Assay

HEK293T or *α*345(IV)-G1244D stable cells were plated on 12-well plates and transiently transfected with plasmid DNAs using TransIT LT-1 reagent (Mirus) or with 15 nM si-RNA using Lipofectamine RNAiMax (Thermo Fisher, MA). The sequences of si-RNAs are listed in Table 1. After 24 hours, cells were replated on 96-well white/clear bottom plates (Corning) and treated. Cells and media were assayed as above.

**Table 1:** Sequences of si-RNA oligonucleotides

Gene Name	Sense (5′–3′)	Antisense (5′–3′)
*PPIA*	CAAGAUGACUAAUGUCAAA	UUUGACAUUAGUCAUCUUG
*PPIB*	GGUCUCUUCGGAAAGACUGUUCCAA	UUGGAACAGUCUUUCCGAAGAGACC
*PPIC*	GCAACAGGAGAGAAAGGAUAUGGAU	AUCCAUAUCCUUUCUCUCCUGUUGC
*PPID*	GCCAAGUAAUUAAAGGAAUAGGAGU	ACUCCUAUUCCUUUAAUUACUUGGC
*PPIE*	CCAAUGAGAAUUAAGGAAGGCUCUU	AAGAGCCUUCCUUAAUUCUCAUUGG
*PPIF*	GGCAGAUGUCGUCCCAAAG	CUUUGGGACGACAUCUGCC
*PPIG*	GGGAUAAGAGUGAGUUGAAUGAAAU	AUUUCAUUCAACUCACUCUUAUCCC
*PPIH*	CGUGGUGUUCUUUGAUGUCAGUAUU	AAUACUGACAUCAAAGAACACCACG
*PPIL1*	GAACUUUGCUGAGUUGGCUCGUCGA	UCGACGAGCCAACUCAGCAAAGUUC
*NKTR*	GGAAAGCAGCAUGUCCGAAAGUAAA	UUUACUUUCGGACAUGCUGCUUUCC
*PPWD1*	GACCCAACAAUAGUCUGUACAUCAU	AUGAUGUACAGACUAUUGUUGGGUC

### Statistical Analysis

Statistical parameters are indicated in the figure legends. Assays were performed in triplicate. All data are presented as mean±SEM. The significance of differences among groups was assessed using ANOVA with Dunnett or Tukey–Kramer tests. Differences with *P* values <0.05 were considered statistically significant.

## Results

### Screening of Natural Product Extract Libraries Revealed That CsA Can Enhance the Secretion of *α*345(IV) Mutant Trimer

**Figure 1:**
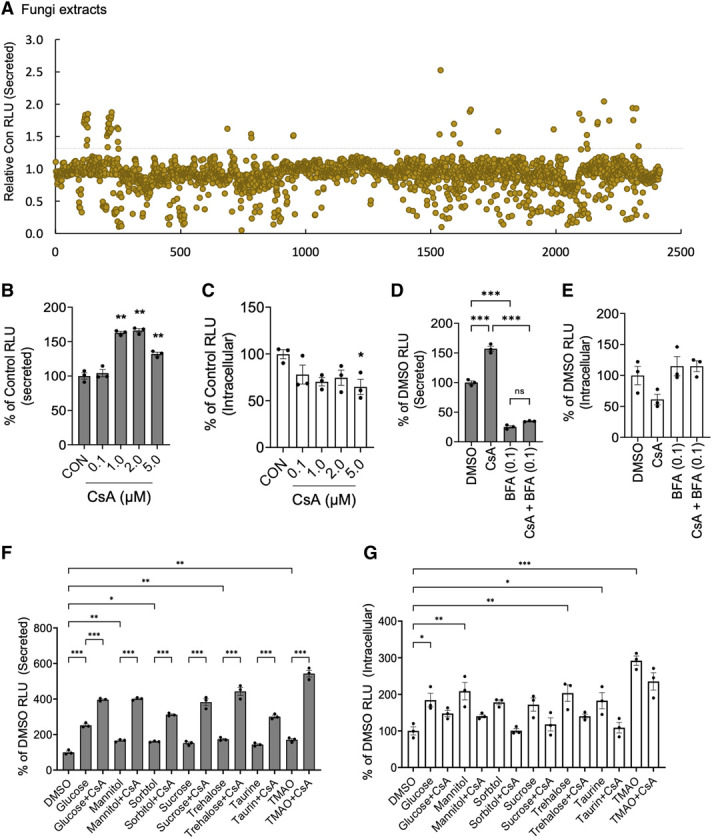
**Screening of natural product extract libraries revealed that CsA can promote the secretion of** *α***345(IV) mutant trimer.** (A) HEK293T cells stably expressing *α*345(IV)-G1244D were treated for 24 hours with a library of fungi extracts. Luminescence of secreted *α*345(IV) trimer in culture medium was measured and expressed as % of control RLU (DMSO). The threshold for positive extracts was set at 130% of control RLU (1.3-fold) indicated by the dotted line. (B–E) *α*345(IV)-G1244D stable cells were treated for 24 hours with the indicated dose of CsA (B and C) or with 1 *μ*M CsA and/or 0.1 *μ*g/ml BFA (D and E). (F and G) Stable cells were treated for 24 hours with 1 *μ*M CsA and/or 150 mM of the indicated osmolyte. Luminescence of secreted and intracellular trimer was measured and expressed as % of control RLU. Data are mean±SEM (*n*=3). *P < 0.05, **P < 0.01, ***P < 0.001 versus control (CON) (DMSO), assessed by ANOVA with the Dunnett test (B and C) or Tukey test (D–G). RLU, relative light unit.

Among the *α*5(IV) missense mutants, we selected G1244D mutant as a representative of mutants that can form trimers intracellularly but have secretion defect.^13^ We chose this mutant because we have AS patient-derived induced pluripotent stem cells with *α*5(IV) G1244D mutation, which could be used to assess the results from the screening in further studies (unpublished data). We validated the *α*345(IV)-G1244D stable cells in 96-well format for luminescence-based HTS. It satisfied the acceptance criteria for HTS^18^ with a Z-factor of 0.67 (Supplemental Figure 1A). To specify a threshold value of hit candidates, we first treated the cells with 150 mM TMAO, one of the chemical chaperones that increase the secretion of mutant *α*345(IV) trimer.^13^ TMAO increased the *α*345(IV)-G1244D trimer secretion to 130% relative light unit compared with DMSO treatment (control; Supplemental Figure 1B). Therefore, we set the hit line at 130% or 1.3-fold increase over control. Next, we screened a library of compounds (635) and more than 13,000 natural product extracts from mushrooms (320), marine sponges (200), plants (2,415), fungi (2,415), and bacteria (7,689) (Supplemental Figure 2). From the screening, 34 hit extracts were obtained, all of which were from fungi (Figure 1A). These hit extracts were analyzed by HPLC-MS/MS (data not shown). Interestingly, the major compound commonly contained in these extracts was CsA, so we focused on its effect on mutant trimer.

Cells were treated with CsA at various concentrations, and the luminescence levels of intracellular and secreted trimer were measured. CsA increased the secretion of mutant trimer within the concentration range of 1–5 *μ*M (Figure 1B). By contrast, CsA treatment decreased the intracellular level of *α*345(IV)-G1244D mutant trimer (Figure 1C). These results suggested that CsA promoted mutant trimer secretion but may not induce intracellular trimerization. To examine this point, cells were treated singly or cotreated with CsA and BFA, an inhibitor of ER to Golgi protein transport and secretion. BFA notably suppressed the CsA-induced increase in trimer secretion (Figure 1D). Within the cells, the level of mutant trimer was not increased by the cotreatment with CsA and BFA compared with BFA alone (Figure 1E). These results indicated that the CsA-induced trimer secretion is through conventional ER-Golgi secretory pathway, and blocking this pathway prevented the CsA-induced trafficking of intracellular trimer. Because CsA, in the presence of BFA, did not increase the intracellular trimer level, CsA is unlikely to induce intracellular formation.

We have shown previously that intracellular *α*345(IV) trimerization was increased by osmolytes,^13^ which are chemical chaperones that can enhance protein folding.^19^ To determine the combinatorial effect of osmolytes and CsA, we treated *α*345(IV)-G1244D cells with osmolytes or with CsA+osmolytes. Osmolytes were able to increase both intracellular and extracellular mutant trimers (Figure 1, F,G). However, CsA+osmolytes significantly increased the secreted trimer compared with osmolyte alone (Figure 1F), with consistent decrease in the intracellular trimer (Figure 1G). These data suggest that osmolytes increased the intracellular trimer, likely through better protein folding, and that CsA promoted the secretion of these trimers resulting in a substantial increase in secreted mutant trimer.

## PPIF/CypD is Involved in the CsA-Induced Secretion of Mutant Trimer

**Figure 2:**
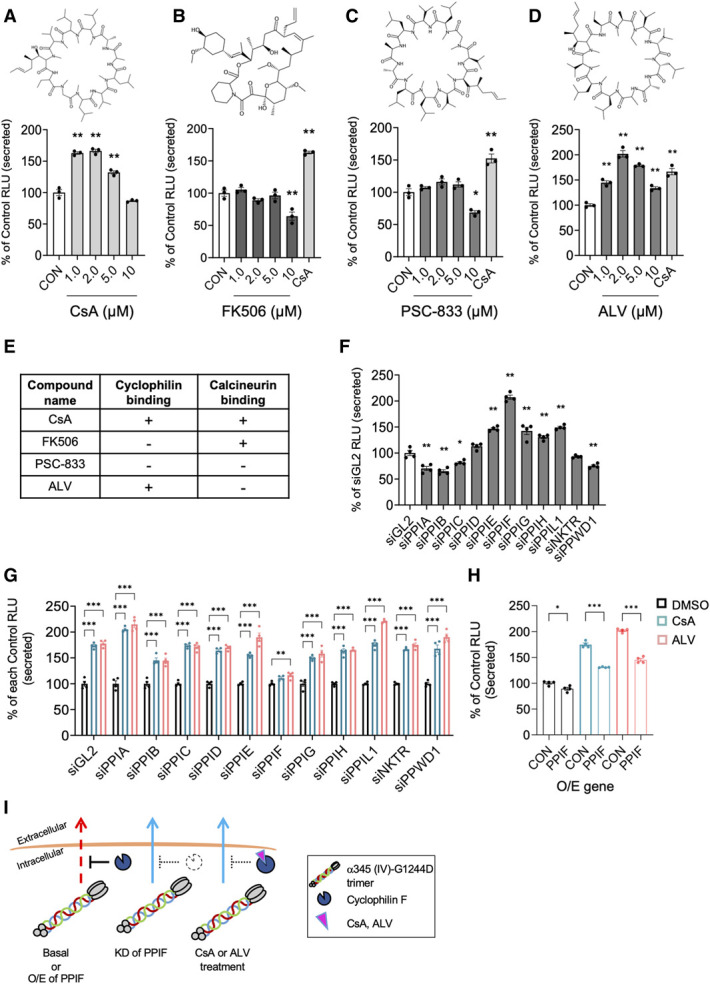
**PPIF/CypD is involved in the cyclosporin-induced secretion of mutant trimer.** (A–D, *lower panels*) *α*345(IV)-G1244D stable cells were treated for 24 hours with the indicated dose of compounds or 1 *μ*M CsA. DMSO was used as control. Luminescence of secreted trimer was measured and expressed as % of control RLU. Data are mean±SEM (*n*=3). *P < 0.05, **P < 0.01 versus CON (DMSO), assessed by ANOVA with the Tukey test. (*Upper panels*) The chemical structures of the compounds are shown; drawn using MarvinSketch (v21.18). (E) Binding ability of compound to cyclophilin and/or calcineurin is indicated. (F–H) Stable cells were transfected with the indicated siRNA oligos (F and G) or with PPIF/CypD-expressing plasmid (H), and replated as described in the Methods. Medium was changed (F), or cells were treated with 1 *μ*M CsA or 2 *μ*M ALV for 24 hours (G and H). Luminescence of secreted trimer was measured and expressed as % of CON (DMSO) RLU. Data are mean±SEM (*n*=3). *P<0.05, **P<0.01, ***P < 0.001; for (F) versus si-GL2 assessed by ANOVA with the Tukey test; for (G) versus control DMSO assessed by ANOVA with the Dunnett test; and for (H) versus control plasmid assessed by the *t* test. (I) Schematic diagram of the regulation of *α*345(IV)-G1244D by PPIF/CypD and CsA, ALV. RLU, relative light unit.

To reveal the mechanism of the effect of CsA, we used structure–activity relationship approach. CsA and FK506 (Tacrolimus) are well-known immunosuppressors through the binding of calcineurin.^20,21^ Therefore, we examined the importance of their calcineurin-binding function. Unlike CsA, FK506 did not increase *α*345(IV)-G1244D trimer secretion (Figure 2, A,B). PSC-833 (valspodar), a CsA derivative that does not bind to cyclophilin and calcineurin,^22,23^ also did not increase the secretion (Figure 2C). Interestingly, ALV/Debio-025,^24^ a CsA derivative that possesses cyclophilin-binding but not calcineurin-binding domain, was able to increase trimer secretion (Figure 2D). These data suggest that the cyclophilin-binding but not calcineurin-binding activity of CsA is critical in its effect of inducing mutant trimer secretion (Figure 2E).

Cyclophilins, encoded by the *PPI* genes, are a family of enzymes with peptidyl prolyl isomerase (PPI) activity, which is responsible for the cis–trans isomerization of peptide bonds *N*-terminal to proline residues.^25^ We investigated which Cyp/PPI isoform is involved in the effect of CsA and ALV by performing knockdown (KD) analysis of known CsA-binding Cyp/PPI genes. The KD efficiency was confirmed by quantitative RT-PCR (Supplemental Figure 3). KD of *PPID* or *NKTR* did not significantly affect the secretion of *α*345(IV)-G1244D trimer, whereas KD of *PPIA*, *PPIB*, *PPIC*, and *PPWD1* slightly decreased the mutant trimer secretion (Figure 2F). By contrast, KD of *PPIE*, *PPIG*, *PPIH*, and *PPIL1* increased the mutant secretion by 1.5-fold, and KD of *PPIF* induced two-fold increase in secretion compared with si-GL2 control (Figure 2F). Among these small-interfering ribonucleic acid (siRNA) nucleotides, only si-*PPIF* inhibited the CsA-induced or ALV-induced increase of mutant trimer secretion (Figure 2G). These data suggested that PPIF/CypD is essentially involved in the trimer secretion-enhancing effects of CsA and ALV. Furthermore, overexpression of PPIF/CypD resulted in the significant decrease of CsA-induced or ALV-induced mutant trimer secretion (Figure 2H). Taken together, these results showed that PPIF/CypD inhibits the secretion of mutant trimer and that this inhibition is alleviated by CsA or ALV treatment (Figure 2I).

## CsA or ALV Promotes the Extracellular Secretion of Some Mutant Trimers

**Figure 3:**
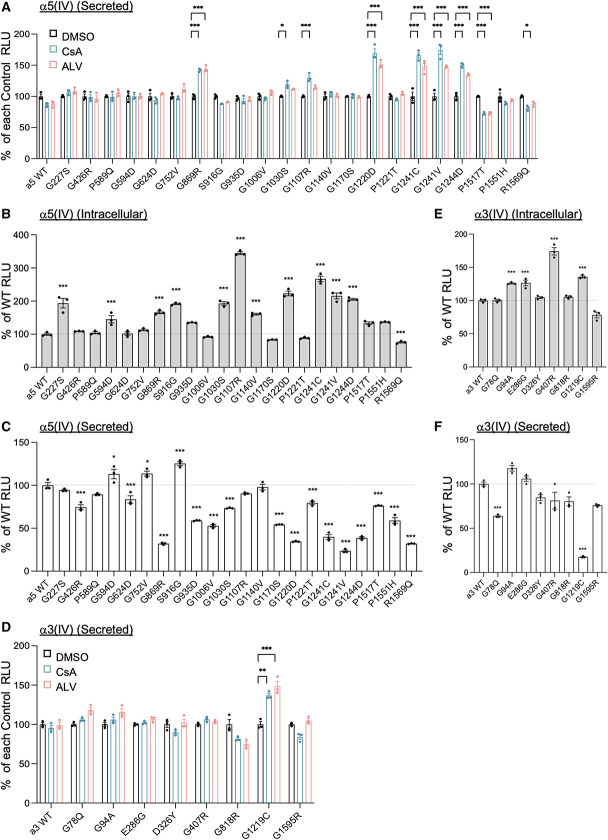
**CsA or ALV promotes the extracellular secretion of some mutant trimers.** (A–F) HEK293T cells were cotransfected with *α*3(IV), *α*4(IV), and *α*5(IV) WT or the indicated *α*5(IV) (A–C) or *α*3(IV) mutants (D–F). After 24 hours, cells were treated with 1 *μ*M CsA or 2 *μ*M ALV (A and D). DMSO was used as control. Luminescence of trimers was measured and expressed as % of control RLU (DMSO of each mutant; A and D) or % of WT (B, C, E, and F). Data are mean±SEM (*n*=3). *P < 0.05, **P < 0.01, ***P < 0.001 versus DMSO (A and D) by ANOVA with the Dunnett test, or WT (B, C, E, and F), assessed by ANOVA with the Tukey test. RLU, relative light unit.

We investigated whether CsA and ALV can increase the trimer secretion of other *α*5(IV) mutants. We examined the clinically reported *α*5(IV) missense mutants of which the trimerization we previously assessed.^10,13^ Compared with DMSO, CsA and ALV markedly increased the secretion of mutants G869R, G1220D, G1241C, G1241V, and G1244D; and slightly increased that of G1030S and G1107R, but not that of *α*345(IV) wild type (WT) and other mutants, indicating that CsA and ALV do not have broad effect on *α*345(IV) trimer secretion (Figure 3A). Except for P1517T and R1569Q, the intracellular trimer levels were not affected by these compounds (Supplemental Figure 4A). There was no discernable pattern of the effect of CsA and ALV on the missense mutants. However, we observed that the mutants that were highly responsive to CsA and ALV could form trimers intracellularly but have defective secretion when compared with *α*345(IV) WT (Figure 3, B,C; intracellular and secreted, respectively).

We next assessed whether these compounds can affect missense mutations in *α*3(IV). We previously examined the trimerization of several *α*3(IV) mutants^10^ and treated them with CsA or ALV. The secretion of *α*3(IV) G1219C mutant was increased by CsA and ALV (Figure 3D), indicating that these compounds can be used not only for *α*5(IV) but also for *α*3(IV) missense mutant. Like the *α*5(IV) mutants that are responsive to CsA and ALV, *α*3(IV) G1219C mutant showed severe secretion defect when compared with WT (Figure 3, E,F). Together, these data indicated that CsA and ALV may promote the secretion of mutant *α*345(IV) trimers that are capable of intracellular trimer formation. CsA and ALV did not affect the intracellular trimer level (Supplemental Figure 4B).

## Discussion

The screening platform that we previously developed was used to screen a library of natural product extracts, and we identified CsA from fungal extracts as a lead compound that increased the secretion of *α*345(IV)-G1244D trimer. Callís *et al.* first reported the positive effects of CsA on proteinuria in patients with AS with a follow-up report of the beneficial effects of long-term treatment.^15,26^ CsA was shown to decelerate the progression of AS in a canine model by delaying the deterioration of the GBM structure.^27^ However, Subsequent clinical studies showed conflicting results of CsA treatment, ranging from effective in reducing proteinuria for short-term use to minimal effect and inducing nephrotoxicity.^16, 28–30^ Owing to concerns of possible nephrotoxic effects with its long-term use, CsA is currently not recommended as therapeutic agent for AS.^16^ Our scrutiny of the structure–activity relationship of CsA revealed that ALV, a derivative of CsA, showed similar effect as CsA on some *α*3(IV) and *α*5(IV) missense mutants. This is promising because ALV was shown to be less toxic than CsA.^31^ ALV does not inhibit calcineurin and has been investigated through phase III trials as treatment for chronic hepatitis C virus.^17,32^

Importantly, the structure–activity relationship analysis uncovered the involvement of cyclophilins, particularly PPIF/CypD, in mediating the effect of CsA and ALV on trimer secretion. Among the cyclophilins, PPIB/CypB is well known to facilitate the folding and hydroxylation of type I collagen.^33–35^ But aside from this, little is known about the role of other PPIs especially in type IV collagen. Here, we found that many of the cyclophilin family members affected the secretion of the mutant *α*345(IV) trimer, with PPIF/CypD causing the biggest change. PPIF/CypD regulates the opening of mitochondrial permeability transition pore and Ca^2+^ homeostasis for optimal metabolic function.^36,37^ PPIF/CypD is mostly known for its function and localization in mitochondria in truncated isoform, but it exists in its full-length form in the cytosol,^38^ the function of which is not well elucidated. *PPIF* gene is distinct from *PPID* gene that encodes CypD/Cyp40, which is known to act as cochaperone in Hsp90 complexes.^39^ Because the KD of other PPIs affected the trimer secretion of *α*345(IV)-G1244D in untreated cells (Figure 2F), it is possible that these PPIs have a role in the regulation of trimer secretion. However, the effect of CsA and ALV was influenced only by PPIF/CypD KD (Figure 2G), making PPIF/CypD the most likely candidate cyclophilin that mediates the effect of CsA and ALV. At present, it is unclear how PPIF/CypD contributes to the modulation of trimer secretion. We were unable to detect direct interaction between *α*5(IV)-G1244D and PPIF/CypD (data not shown). Previous reports showed that CypB forms complexes with other proteins, such as Hsp47, protein disulfide isomerase, and calnexin, to facilitate the hydroxylation and folding of procollagen.^40–42^ Further studies may clarify whether PPIF/CypD associates with other molecular chaperones to modulate the trimer secretion of *α*345(IV)-G1244D. The group by Peterson *et al.* has recently developed selective inhibitors for PPIF/CypD and PPIE/CypE.^43^ Investigating the effect of these inhibitors on *α*345(IV) mutant trimer secretion may be warranted because if effective, these compounds may have less side effects because of its high specificity.

We used osmolytes to enhance protein folding of *α*345(IV) that resulted in increased intracellular trimer. Owing to the broad effects of osmolytes, the *α*345(IV) trimerization was likely a nonspecific effect of the general propensity of osmolytes to fold proteins. Regardless, the data showed that combining the effect of ALV on secretion with a compound that promotes trimerization results in further extracellular secretion and may provide greater therapeutic benefit. At present, there is neither an established criterion for the level of mutant trimer that must be secreted to function similarly to wild type trimer nor an assay to validate the functionality of the secreted mutant trimer. The lack of appropriate mouse model also hinders the *in vivo* testing of CsA and ALV efficacy.

Our current study supports the clinical findings that CsA could be effective, but its effect may be limited to some mutations. We propose that the applicability of CsA and ALV depends on the type of mutation in the collagen component genes, so more data on mutant trimer secretion, correlation with clinical characteristics, and mutants' responsiveness to CsA and ALV should be collected. The number of mutations found in AS is increasing as genetic analysis progresses, and it is not feasible to develop tailor-made drugs for each mutation. Because many of the mutants that showed responsiveness to CsA and ALV could form trimers intracellularly but have <50% basal trimer secretion compared with wild type, it is likely that CsA and ALV are more effective for mutants with secretion defect, but ineffective for mutants that are incapable of forming intracellular trimers. In the clinical study by Masella *et al.*, a patient with AS had the mutation G1107R, which in our system was moderately responsive to CsA or ALV. However, it was unclear how efficiently CsA worked on this mutation clinically.^30^ While data on efficacy against more variants need to be gathered, taken together, our findings suggest that ALV, whose safety profile has been elucidated, would be a potential therapeutic agent that can be presented to patients with AS with ALV-responsive mutations. Moreover, our findings add to our understanding of the fundamental regulation of *α*345(IV), as we have shown that part of the intracellular regulatory mechanism of *α*345(IV) involves the cyclophilins and proposed a candidate therapeutic approach for AS.

## Disclosures

H. Kai reports the following: Research Funding: Grants-in-Aid for Scientific Research from the Ministry of Education, Sciences, Sports, and Culture (MEXT) of Japan. T. Shuto reports the following: C-HAS+ Co., Ltd.; Ownership Interest: C-HAS+ Co., Ltd.; and Research Funding: Grants-in-Aid for Scientific Research from the Ministry of Education, Sciences, Sports, and Culture (MEXT) of Japan. M. Wada reports the following: Consultancy: Eisai Co., Ltd.; Research Funding: Arden More Co., Ltd., EGAO Co., Ltd., and NIPPON AIM Co., Ltd.; and Patents or Royalties: “Prevention or amelioration of metabolic syndrome” JP B1 P6770726. All remaining authors have nothing to disclose.

## Supplementary Material

## Data Availability

All data are included in the manuscript and/or supporting information.

## Supplemental Material

Supplemental Method. Quantitative real-time RT-PCR.

Supplemental Table 1. Sequences of primers used for quantitative real-time RT-PCR.

Supplemental Figure 1. Validation and TMAO treatment of *α*345(IV)-G1244D stable cells.

Supplemental Figure 2. Nanoluciferase-based screening of natural product extracts and compounds for the secretion of *α*345(IV)-G1244D trimer.

Supplemental Figure 3. Quantitative real-time RT-PCR analysis of PPI genes to confirm the efficiency of knockdown by siRNA.

Supplemental Figure 4. Effect of CsA or ALV on the intracellular trimer formation of mutants.
