# Supplementary material for: CyclosporinA Derivative as Therapeutic Candidate for Alport Syndrome by Inducing Mutant Type IV Collagen Secretion
Source: Kidney360. 2023 May 5;4(7):909–17. doi: 10.34067/KID.0000000000000134 (PMC10371266; doi:10.34067/KID.0000000000000134)
Supplement: Supplementary file 1 [file kidney360-4-0909-s001.pdf]

Supplementary Materials
Kuwazuru, J., et al

Contents

Supplementary Method

Quantitative real-time RT-PCR

Supplementary Table 1

Table S1. Sequences of primers used for quantitative real-time RT-PCR

Supplementary Figures

Figure S1. Validation and TMAO treatment of α345(IV)-G1244D stable cells

Figure S2. Nanoluciferase-based screening of natural product extracts and compounds for the secretion of α345(IV)-G1244D trimer

Figure S3.Quantitative real-time RT-PCR analysis of PPI genes to confirm the efficiency of knockdown by si-RNA

Figure S4. Effect of CsA or ALV on the intracellular trimer formation of mutants

Supplementary Method

Quantitative Real-Time PCR

Total RNA was isolated from α345(IV)-G1244D stable cells cells using RNAiso (Takara, Japan) following the recommended protocol. Reverse transcription and PCR amplification were performed using PrimeScript RT Reagent Kit and SYBR Premix ExTaq II (Takara) as described previously (Suico, et al., JBC 2014). The sequences of primers used for real-time qPCR are listed in Table S1.

Supplementary Table

Table S1. Sequences of primers used for quantitative real-time RT-PCR

gene	Forward (5' to 3')	Reverse (5' to 3')
PPIA	CACTGTTGATGTTCTTGAGGGAAG	AGGCTCTATATGCTACAAGCAGTAC
PPIB	TCCAGGGCGGAGACTTCAC	ACTCCTTGGCGATGGCAAAG
PPIC	AGCAAGTTTCATCGTGTCATCA	TGGAAATGTCTCACCATAGATGC
PPID	TGGACACACGACTGGGAAAC	AAACCCTCCCGATCATGCTTG
PPIE	GCTTTTTGGACGTACAATTCGTG	TCTTCAAGCGTCTTCCCAGAA
PPIF	GAAGGCAGATGTCGTCCCAAA	GGAAAGCGGCTTCCGTAGAT
PPIG	ACTCCTCATTTAGATGGGCATCA	GAGTATCCGTACCTCCGCAAA
PPIH	TTTGCAGACGTTGTGCCTAAG	CCTTTGTATCCTATTGGAACCCC
PPIL1	TGAGTTGGCTCGTCGAGGTTA	GCCATAGATAGATGCACCACC
NKTR	GAGCCGGTTGGTCGCATTAT	ACACGATGGAACGTAGAACCTT
PPWD1	TGGCCGAGGAGATAACCAG	CCCAGTCCAGTATTCAATCATCC

Supplementary Figures

Supplementary Figure 1.

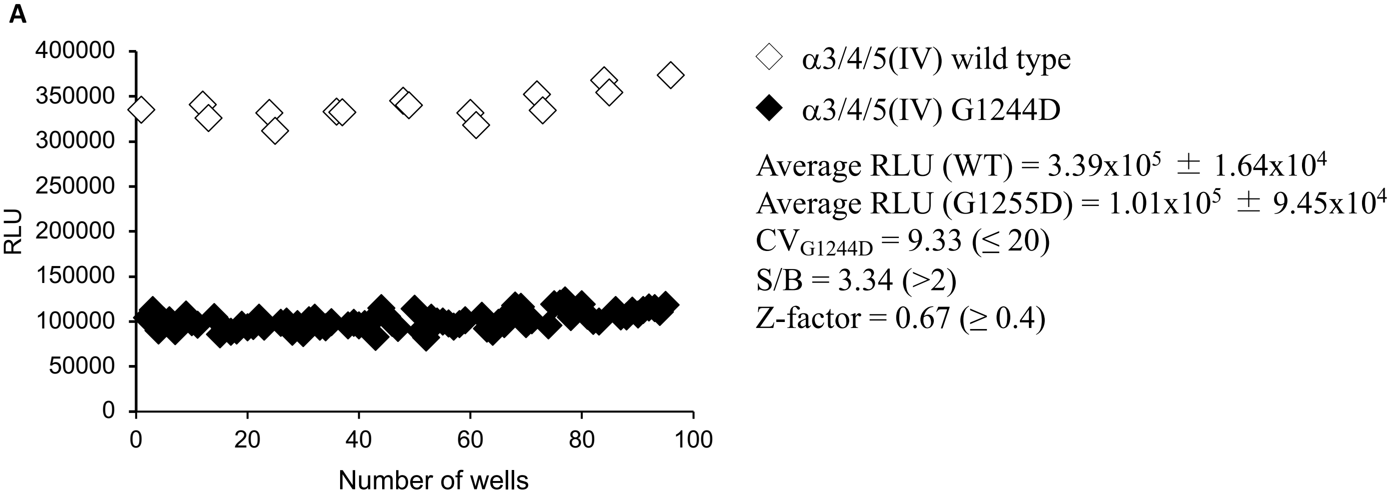

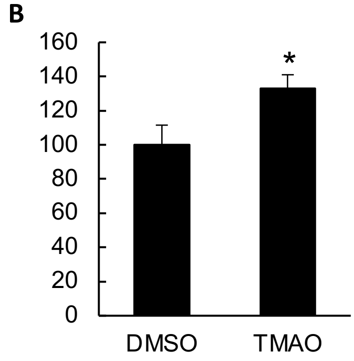

Figure S1. Validation and TMAO treatment of α345(IV)-G1244D stable cells

(A) α345(IV)-WT (n=16) and α345(IV)-G1244D (n=80) stable cells were plated in 96-well white plate. Twenty four hr after plating, medium was changed with fresh medium containing 200 μM ascorbic acid, and cells were incubated for additional 24 hr. Medium was assayed for nanoluciferase activity. (B) α345(IV)-G1244D stable cells were plated in 96-well white plate. At sub-confluent, cells were treated with DMSO (1%) or 150 mM TMAO for 24 hrs. Luminescence of medium was measured. Data are presented as mean ± SE (n=3 per group). P values were assessed by Student's t test. (*$p<0.05$).

Supplementary Figure 2.

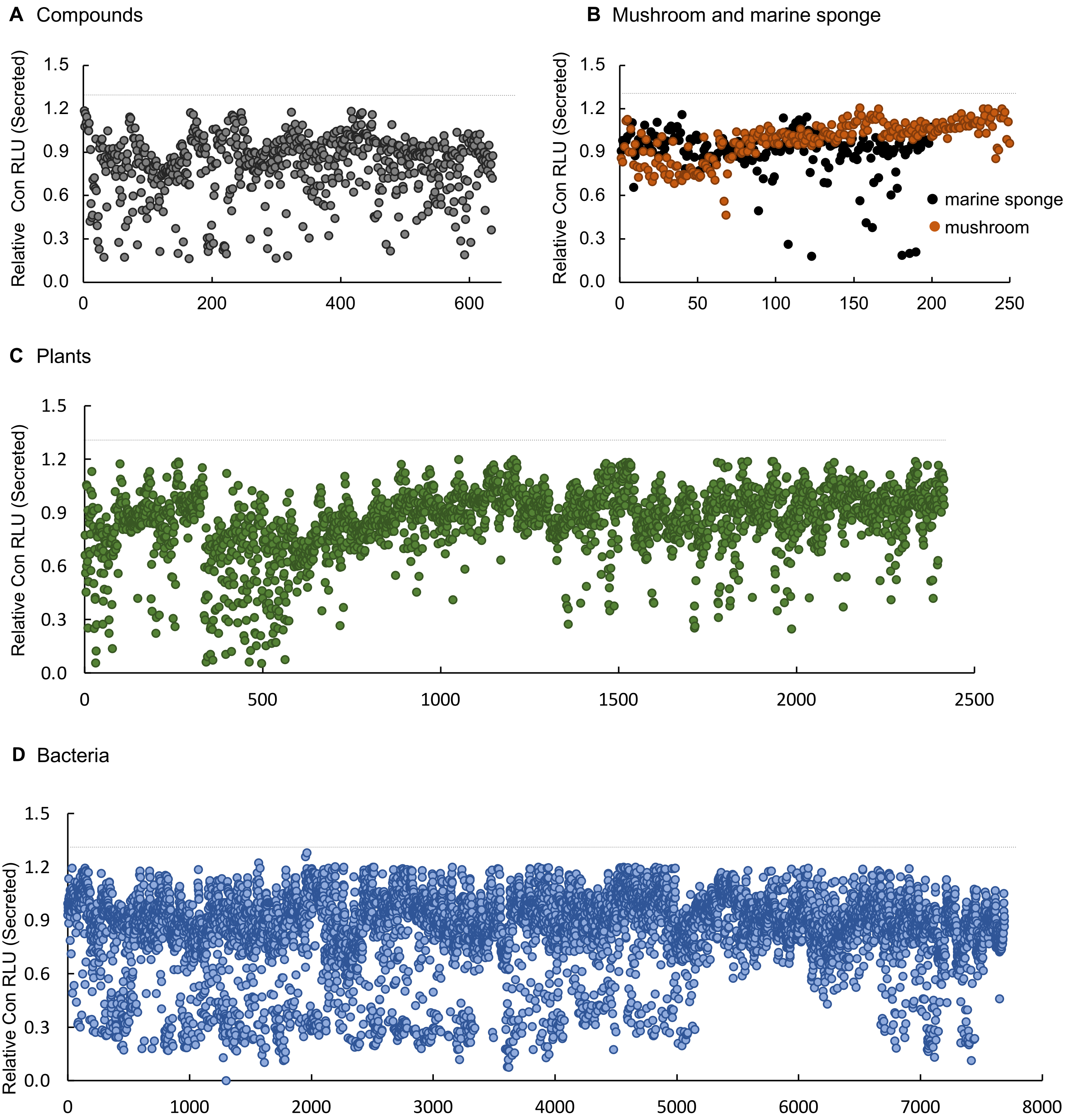

Figure S2. Nanoluciferase-based screening of natural product extracts and compounds for the secretion of α345(IV)-G1244D trimer.
(A-D). HEK293T cells stably expressing α345(IV)-G1244D were treated for 24 hr with compounds or natural product extracts. Luminescence of secreted α345(IV) trimer in culture medium was measured and expressed as % of control RLU (DMSO). The threshold for positive extracts was set at 130% of control RLU (1.3-fold) indicated by the dotted line.

Supplementary Figure 3.

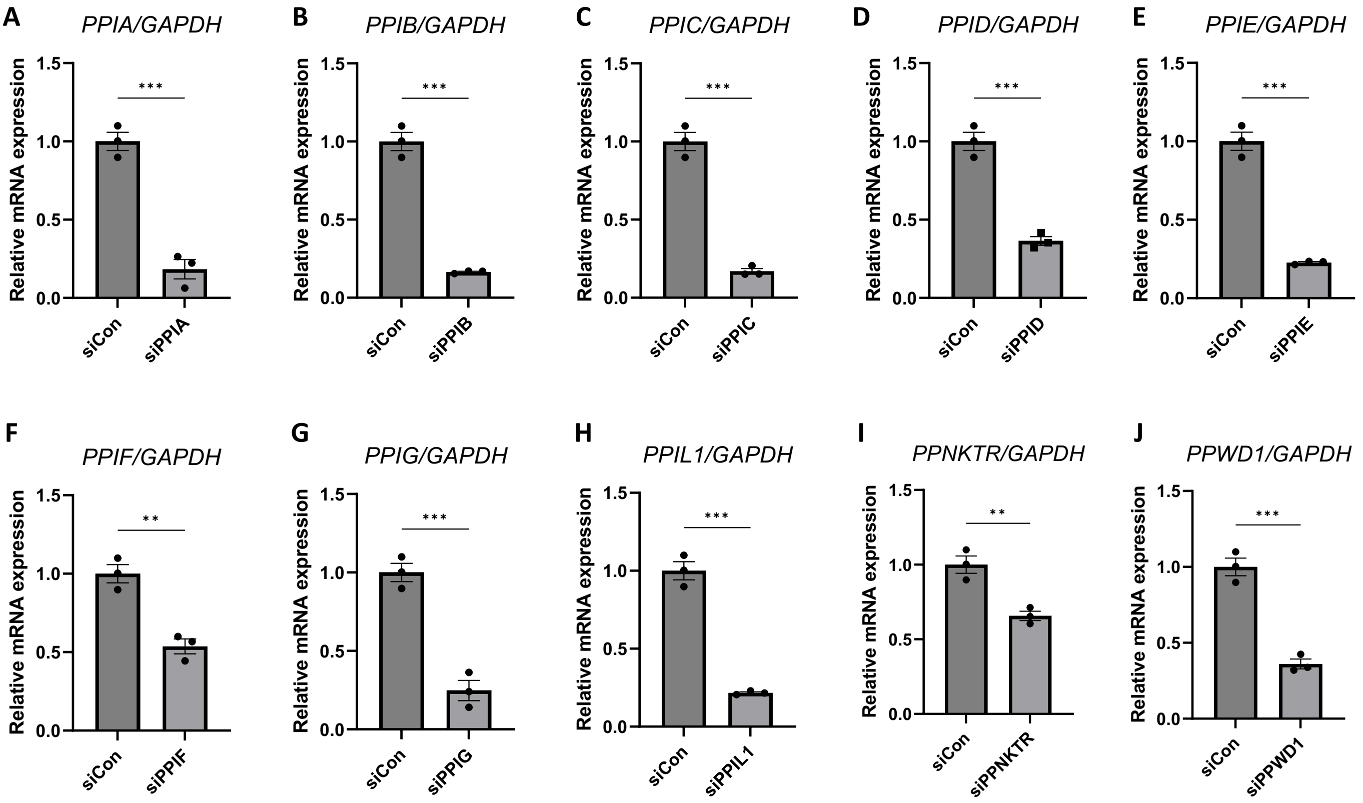

Figure S3. Quantitative real-time RT-PCR analysis of PPI genes to confirm the efficiency of knockdown by si-RNA

(A-J) Total RNA was isolated from cells as described in Supplementary Methods. Gene expression of PPIs were analyzed to confirm the efficiency of siRNAs. Data are presented as mean ± SD (n=3 per group). P values were assessed by Student’s t test. (**p<0.01, ***p<0.001).

Supplementary Figure 4.

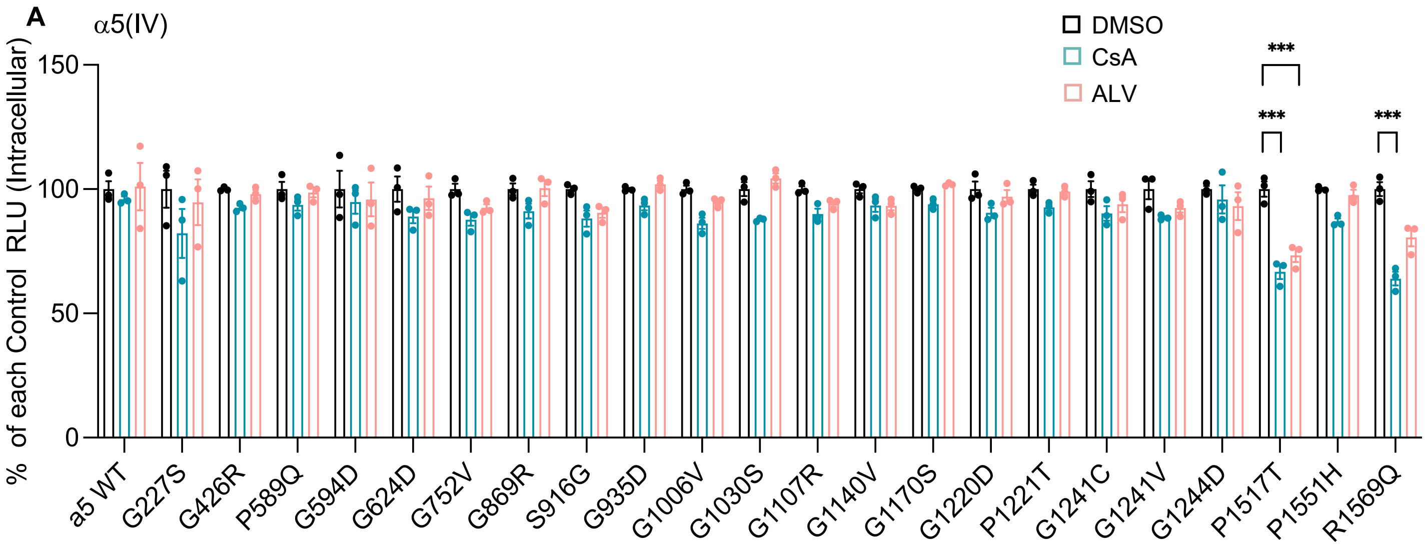

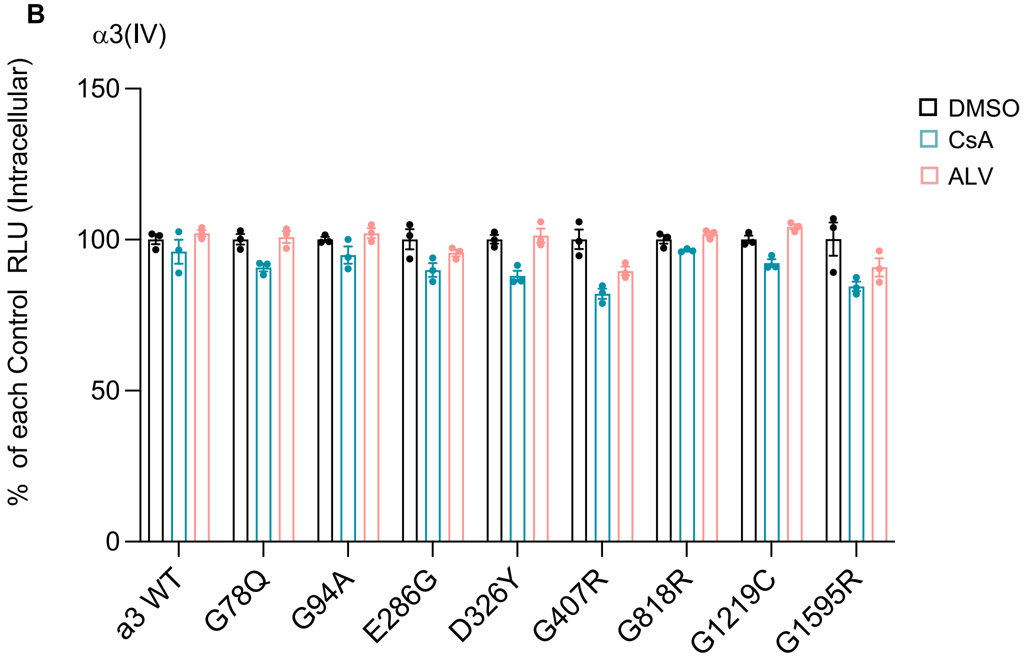

Figure S4. Effect of CsA or ALV on the intracellular trimer formation of mutants.
HEK293T cells were co-transfected with wild-type α3(IV), α4(IV), α5(IV) or the indicated mutant α5(IV) (A), or α3(IV) (B). After 24 hr, cells were treated with 1 μM CsA or 2 μM ALV. DMSO was used as control. Luminescence of intracellular trimer was measured. Data are mean ± S.E (n=3). ***P<0.001 versus DMSO, assessed by ANOVA with Tukey test.
